# Arthroscopic superior capsule reconstruction with dermal allograft and autologous long head of the biceps tendon for irreparable posterosuperior rotator cuff tears, a two-year clinical and radiological results

**DOI:** 10.1016/j.clinsp.2025.100628

**Published:** 2025-04-02

**Authors:** Joe Chih-Hao Chiu, Yu-Cheng Chen, Poyu Chen, Yi Lu, Cheng-Pang Yang, You-Hung Cheng, Alvin Chao-Yu Chen

**Affiliations:** Department of Orthopedic Surgery, Chang Gung Memorial Hospital, Linkou, Taiwan

**Keywords:** Dermal allograft, Long head of biceps, Rotator cuff, Superior capsule reconstruction

## Abstract

**Background:**

A 2 mm-thick dermal allograft and autologous Long Head of the Biceps Tendon (LHBT) Superior Capsule Reconstruction (SCR) could provide improved clinical and radiographic outcomes for posterosuperior Massive Irreparable Rotator Cuff Tears (MIRCTs).

**Methods:**

A retrospective study was conducted between April 2019 and October 2021. The LHBT was rerouted 5 mm posteriorly to the bicipital groove, and a 2 mm-thcick dermal allograft was used to cover the rerouted LHBT. Clinical assessment included Constant-Murley Shoulder score (CMS), American Shoulder and Elbow Surgeons (ASES) score, Subjective Shoulder Value (SSV), Visual Analog Scale (VAS) pain scores, and active ROM measurements. The radiological assessment included Acromiohumeral Distance (AHD), Superior Capsular Distance (SCD), muscle Fatty Infiltration (FI), and follow-up SCR integrity using ultrasound two years after surgery.

**Results:**

25 patients (7 male, 18 female) were included with a mean age of 64.2 ± 6.9 years. Active ROM, AHD, and SCD did not change significantly after the surgery. Patients' VAS (8.3 ± 0.7 to 1.3 ± 0.6, *p* < 0.001), SSV (22.4 ± 8.6 to 77.6 ± 12.7, *p* < 0.001), CMS (36.2 ± 6.8 to 79.9 ± 8.4, *p* < 0.001), and ASES (37.2 ± 10.0 to 80.5 ± 5.7, *p* < 0.001) improved significantly at final follow-up. The ultrasound examination of all patients demonstrated a healed dermal allograft on the supraspinatus footprint. One patient (4 %) had a postoperative trauma causing irreparable subscapularis tear, leading to reverse total shoulder arthroplasty during revision surgery.

**Conclusions:**

The combined SCR technique using a 2 mm dermal allograft and autologous LHBT for posterosuperior MIRCTs significantly improved the patient-reported outcomes.

## Highlights

• Superior capsule reconstruction with an autologous iliotibial band, dermal allograft, and long head of biceps tendon has been proposed to treat massive irreparable posterosuperior rotator cuff tears.
• Superior capsule reconstruction with dermal allograft combined with autologous long head of biceps tendon demonstrates significant improvements in shoulder function and pain relief over two years for patients with irreparable posterosuperior rotator cuff tears.
• The indications for this method included painful irreparable posterosuperior massive rotator cuff tears less or equal to Hamada grade 2 rotator cuff tear arthropathy, supraspinatus tendon retraction at Patte stage III, no preoperative pseudoparesis, supraspinatus fatty infiltration equal to or more than Goutallier stage 2, and presence of long head of the biceps tendon.

## Introduction

Mihata et al.^1^ initially reported on Superior Capsule Reconstruction (SCR) using autologous fascia lata in their study addressing irreparable Rotator Cuff Tears (RCTs). The technique has evolved to incorporate various modifications, including the use of dermal allografts,^2^^,^^3^ Long Head of the Biceps Tendon (LHBT),^4^^,^^5^ and combinations of dermal allografts and autologous LHBT^6^ with different thicknesses, configurations, and biomechanical properties. As per the initial design outlined by Mihata et al.,^7^ a 6‒8 mm-thick graft was created by folding the fascia lata two or three times and suturing around the edge of the folds. Denard et al. utilized 1 to 3 mm-thick acellular dermal allografts in their SCR procedures, achieving an 80 % success rate with a 19 % short-term revision rate.^2^ Regarding the LHBT, the average thickness was 6 mm, which was similar to the 6 to 8 mm autologous fasia lata used by Mihata et al.^5^ Barth et al. demonstrated that SCR with LHBT autografts prevents infraspinatus retears in cases of massive posterosuperior RCTs.^4^ Kim et al. compared autologous LHBT with dermal allografts and found no differences between the two groups at two years postoperative, except for the autograft being thicker.^8^

The purpose of this study is to present the two-year results of arthroscopic SCR with a 2 mm-thick dermal allograft and autologous LHBT for irreparable posterosuperior RCTs. The authors hypothesized that this combined SCR approach, utilizing a 2 mm dermal allograft and autologous LHBT, would yield feasible clinical and radiographic outcomes at the two-year follow-up for irreparable posterosuperior RCTs.

## Material and methods

### Study design

This retrospective study was approved by the institution's ethics committee, and informed consent was obtained from all patients. The authors included patients receiving SCR using dermal allograft in conjunction with autologous LHBT between April 2019 and October 2021 by a single surgeon. The indications for this method included painful irreparable posterosuperior massive RCTs less or equal to Hamada grade 2 RCTs arthropathy, supraspinatus tendon retraction at Patte stage III, no preoperative pseudoparesis, supraspinatus Fatty Infiltration (FI) equal to or more than Goutallier stage 2, and presence of LHBT.^6^ Exclusion criteria were previous ipsilateral shoulder surgery, subscapularis tear > Lafosse type 3,^9^ fractures, infection-related pathologies, a partial or complete tear of LHBT, severe glenohumeral joint osteoarthritis, and deformity of the humeral head on preoperative X-Ray. Before the operation, patients' fundamental clinical data was recorded, including sex, age, and the presence of chronic or systemic diseases.

### Surgical techniques

**Fig. 1:**
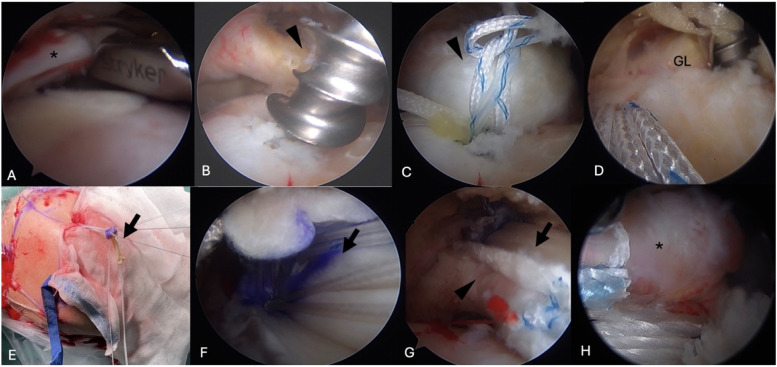
Right shoulder, viewed from lateral portal. (A) The remnant cuff (asterisk) cannot be restored to the anatomical footprint of supraspinatus. (B) A double-loaded, suture-based anchor is inserted 5‒8 mm posterior to the bicipital groove to secure the LHBT (arrowhead) initially. (C) Lasso loops are created through LHBT to provide a posteriorly rerouted LHBT. (D) Two double-loaded anchors are inserted at the glenoid side. (E) A 2 mm-thick dermal allograft (arrow) is introduced into the joint. (F) The sutures from the glenoid anchors are secured. (G) The positioning of the dermal allograft can be covered on top of the LHBT to increase the spacer effect (Fig. 1G). (H) The remaining portions of the supraspinatus and infraspinatus can be repaired through the Swivelock anchor. GL, Glenoid.

The surgical technique involving combined autogenous LHBT and dermal allograft SCR has been previously documented.^6^ In summary, after making sure the remnant cuff cannot be restored to the anatomical footprint (Fig. 1A) of the supraspinatus, a double-loaded or triple-loaded, suture-based anchor is inserted 5‒8 mm posterior to the bicipital groove to secure the LHBT initially (Fig. 1B). Two lasso loops are created through the LHBT before a complete release of the transverse humeral ligament and tenotomy of the LHBT distal to the fixation point are performed, resulting in a posteriorly rerouted LHBT (Fig. 1C). No additional tenodesis is conducted concerning the distal portion of the LHBT. Preservation of the proximal attachment of the biceps on the glenoid side is maintained, ensuring native fixation. Subsequently, a 2 × 4 cm dermal allograft with a thickness of 2 mm (LifeNet Health OrACELL, Virginia Beach, VA) is utilized to cover the rerouted LHBT, enhancing its strength and providing a tensile effect. Four anchors are employed for fixation: two double-loaded anchors at the glenoid side (Fig. 1D) and two BioComposite SwiveLock C anchors (Arthrex, Naples, FL) at the greater tuberosity. Following the introduction of the dermal allograft into the joint (Fig. 1E), the sutures from the glenoid anchors are secured (Fig. 1F), and the optimal tension of the allograft is gauged during the insertion of the lateral Swivelock anchor at 45° shoulder abduction.^1^ The dermal allograft can cover on top of the LHBT to increase the spacer effect (Fig. 1G). Medial row anchors are not necessary. The remaining portions of the supraspinatus and infraspinatus can be repaired using sutures passed through the Swivelock anchor to enhance stability (Fig. 1H). The subscapularis is repaired without the need for additional fixation with the SCR graft.

### Clinical and radiographic assessment

#### Clinical assessment

All patients underwent a detailed physical examination at the outpatient department preoperatively and at the two-year follow-up by an independent orthopedic surgeon blind the information who had >10 years of clinical practice including (1) Constant-Murley Shoulder score (CMS),^10^ (2) American Shoulder and Elbow Surgeons (ASES) score,^11^ (3) Subjective Shoulder Value (SSV),^12^ (4)Visual Analog Scale (VAS, 10 point grading system) pain scores, and, (5) Active ROM including active Forward Flexion (FF), External Rotation by the side (ER1), and Internal Rotation (IR).^13^

#### Radiological assessment

Standard anteroposterior radiography was performed to assess the AHD,^3^ Superior Capsular Distance (SCD),^3^ and Hamada classification^14^ of the glenohumeral joint before surgery and at the two-year follow-up. In all patients, rotator cuff integrity was evaluated before index surgery using 1.5 Tesla Magnetic Resonance Imaging (MRI). The Goutallier classification, as modified by Fuchs et al.^15^ was used to assess supraspinatus and infraspinatus muscle FI. The presence of the tangent sign, retraction, thickness, and length of the supraspinatus tendon, and integrity of the LHBT were also assessed.^16^

All patients underwent routine ultrasound evaluation of their operated shoulder at 12- and 24-months postoperatively. Ultrasound evaluation of the repaired rotator cuff and SCR integrity was conducted by an experienced orthopedic physician who had >10-years of clinical practice and specialized in shoulder pathology and ultrasonography. Structural integrity was evaluated at the two-year follow-up based on the Sugaya classification.^17^

### Statistical analysis

Statistical analyses were performed using SPSS software (version 25.0; IBM, Armonk, NY). The means, standard deviations, and ranges were calculated. Pearson's Chi-Square or Fisher's exact test was used to compare categorical variables. A *t*-test or Wilcoxon rank sum was performed to analyze the difference in preoperative and postoperative outcome scores for ROM, CMS, ASES, SSV, and VAS scores. Two-tailed p-values of <0.05 were considered significant. The authors calculated the Minimal Clinically Important Difference (MCID) for CMS, ASES, SSV, and VAS scores using pre- and post-treatment data. Relevant variables were extracted, and paired *t*-tests were conducted. Cohen's *d* was computed for each variable to determine effect size. The MCID was then calculated by multiplying Cohen's d with the pre-treatment standard deviation of each variable. The resulting MCIDs were CMS = 28.60, ASES = 34.32, SSV = 33.86, and VAS = 6.85. These values represent the threshold for clinically significant changes in each respective score.

## Results

**Fig. 2:**
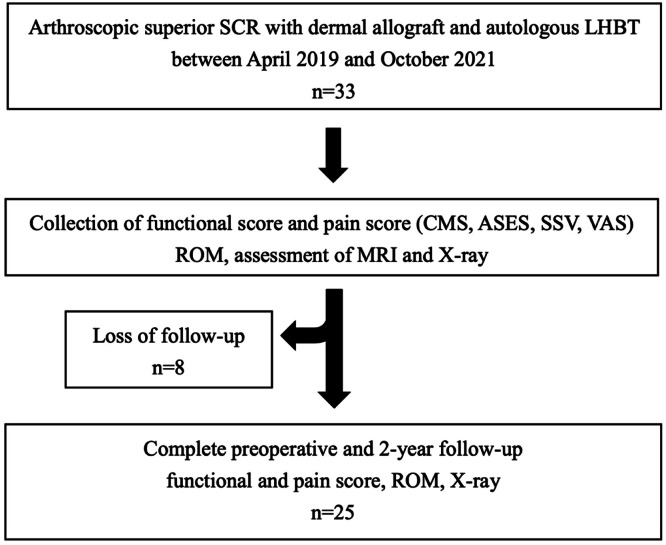
Patient screening flowchart. SCR, Superior Capsule Reconstruction; LHBT, Long Head of the Biceps; ROM, Range of Motion; SSV, Subjective Shoulder Value; ASES, American Shoulder, and Elbow Surgeons; CMS, Costant-Murley Score, VAS, Visual Analog Scale.

A total of 33 patients met the inclusion criteria, and 8 were excluded, leaving 7 male and 18 female for the study (Fig. 2). The patient demographics are listed in Table 1.

**Table 1:** Characteristics of patients.

N° of patient	25
Age (yr)	64.2 ± 6.9
Male / Female	7 / 18
Body mass index (kg/m^2^)	23.5 ± 3.7
Follow up duration (months)	26.2 ± 4.3
Side of surgery, right / left	23 / 2
Systemic disease, (diabetes, hypertension), n ( %)	13 (52 %)
Preoperative X-Ray	
AHD (mm)	7.9 ± 2.5
SCD (mm)	35.4 ± 4.9
Preoperative MRI	
SSP tendon length^31^ (mm)	20.4 ± 8.2
SSP tendon thickness (mm)	4.4 ± 1.7
SSP retraction, Patte classification	
Ⅰ / Ⅱ / Ⅲ (n)	0 / 0 / 25
Fatty change grade (0 / 1 / 2 / 3 / 4)	
SSC	8 / 13 / 3 / 1 / 0
SSP	0 / 0 / 2 / 13 /10
ISP	4 / 13 / 8 / 0 / 0
Preoperative, shoulder Active ROM	
FF (°)	136.8 ± 38.4
ER 1 (°)	62.4 ± 13.0
IR (1∼18)	10.4 ± 3.4

ROM, Range of Motion; AHD, Acromiohumeral Distance; SCD, Superior Capsule Distance; SSV, Subjective Shoulder Value; ASES, American Shoulder and Elbow Surgeons; CMS, Costant-Murley Score, VAS, Visual Analog Scale; FF, Forward Flexion, ER, External Rotation; IR, Internal Rotation, SSP, Supraspinatus, SSC, Subscapularis, ISP, Infraspinatus.

There was no significant change regarding active ROM, AHD, and SCD after the surgery. There was a significant improvement in pain scales and functional outcomes at the 2-year follow-up. The VAS demonstrated substantial improvements, decreasing from 8.3 ± 0.7 to 1.3 ± 0.6, SSV improved from 22.4 ± 8.6 to 77.6 ± 12.7, CMS from 36.2 ± 6.8 to 79.9 ± 8.4, and ASES scores from 37.2 ± 10.0 to 80.5 ± 5.7 at the final follow-up (all *p* < 0.001) (Table 2). All but one patient reached MCIDs for CMS, ASES, SSV, and VAS. All 25 patients demonstrated a healed dermal allograft on the supraspinatus footprint during the follow-up ultrasound examination. One (4 %) patient experienced a retear of the previously Lafosse type 3 subscapularis repair with preserved dermal allograft and LHBT SCR on follow-up MRI. He underwent a Reverse Total Shoulder Arthroplasty (RSA) during the revision surgery, leading to an uneventful recovery.

**Table 2:** Postoperative outcomes.

	Preoperative	Postoperative	p-value
X-Ray			
AHD (mm)	7.9 ± 2.5	8.0 ± 2.0	0.922
SCD (mm)	35.4 ± 4.9	36.0 ± 4.7	0.488
Active ROM			
FF (°)	136.8 ± 38.4	148.0 ± 19.0	0.187
ER 1(°)	62.4 ± 13.0	61.6 ± 10.8	0.784
IR	10.4 ± 3.4	11.1 ± 3.2	0.302
Pain intensity			
VAS	8.3 ± 0.7	1.3 ± 0.6	<0.001
Functional score			
SSV	22.4 ± 8.6	77.6 ± 12.7	<0.001
CMS	36.2 ± 6.8	79.9 ± 8.4	<0.001
ASES	37.2 ± 10.0	80.5 ± 5.7	<0.001

ROM, Range of Motion; AHD, Acromiohumeral Distance; SCD, Superior Capsule Distance; SSV, Subjective Shoulder Value; ASES, American Shoulder and Elbow Surgeons; CMS, Costant-Murley Score, VAS, Visual Analog Scale; FF, Forward Flexion, ER, External Rotation; IR, Internal Rotation.

## Discussion

The study confirmed the hypothesis that the combined SCR technique using a 2 mm dermal allograft and autologous LHBT for irreparable posterosuperior RCTs provided a feasible 2-year clinical and radiological outcome.

Achieving successful SCR hinges on appropriate patient selection, graft coverage, and thickness. While SCR is often indicated for massive rotator cuff tears (2‒3 tendons) and significant cuff retraction (Patte stage 3),^18^ more precise indications, as highlighted by Werthel et al.’s review, suggest SCR for irreparable supraspinatus tears in patients without glenohumeral osteoarthritis (Hamada < 4) and without an associated subscapularis tear. In the present study, the authors specifically enrolled patients with irreparable posterosuperior massive RCTs of Hamada grade 2 or less, ensuring good preoperative active ROM without an ER lag sign and the absence of pseudoparalysis or pseudoparesis. All subscapularis tears in the cohort were reparable during the surgery. In cases with an ER lag sign or pseudoparesis, the authors opted for Lower Trapezius Transfer (LTT) instead of SCR, as LTT may provide a more robust axial plane force couple and greater ER torque than SCR.^19^

Regarding graft coverage and thickness, the native superior capsule of the glenohumeral joint works as a fibrous roof and provides complete coverage in only 27 % of the cases analyzed by Pouliartet at al.^20^ Also, it is a relatively thin fibrous structure that is attached to 30 % to 61 % of the greater tuberosity. The thickness of the superior capsule varies on average between 0.8 mm laterally and 1.2 mm next to the tendons of the supraspinatus and infraspinatus; the thickness is 0.9 mm next to the middle part of the supraspinatus tendon, and its insertion at the level of the glenoid labrum is only 0.6 mm-thick on average. Therefore, it is arguable to claim that Mihata's 6‒8 mm-thick graft^7^ restores the anatomical superior capsule. On the other hand, a good spacer effect and a reverse trampoline effect are mandatory to restore the superior stability of the glenohumeral joint.^7^^,^^20^ For autologous fascia lata, an SCR using at least 6 mm-thick fascia lata significantly increased AHD postoperatively and restored shoulder function.^21^ For dermal allografts, an SCR graft thickness of <3 mm carries an increased risk of clinical and radiologic failure.^22^ The combined technique utilized a 2 mm dermal allograft alongside autologous LHBT. The LHBT alone, with an average thickness of 6.6 mm at the articular margin,^23^ provides a significant spacer effect, while the addition of a dermal allograft enhances superior stability and coverage, potentially preventing joint fluid extravasation and aiding cuff healing.^24^ The authors also preserved the glenoid insertion of the LHBT to mitigate common failures associated with graft tears on the glenoid side, which may be higher for allografts when compared with autografts.^25^

The present study did not show significant improvements in AHD (from 7.9 ± 2.5 mm to 8.0 ± 2.0 mm, *p* = 0.92), which is similar to the results of SCR with dermal allograft by Denard et al.^2^ (AHD from 6.6 ± 3.0 mm to 6.7 ± 3.0 mm, *p* = 0.889). In this series, the authors used a dermal allograft of 2 mm, compared to Denard et al.’s graft (from 1 to 3 mm). Both studies provided successful outcomes in approximately 70 % of cases. This might be due to the inclusion criteria of our SCR, which was irreparable posterosuperior massive rotator cuff tears with Hamada grade 2 or less, indicating a minimal proximal migration and a relatively stable humeral head.

The qualitative assessment, focusing on VAS pain score and shoulder functional scales (ASES, CMS, and SSV), demonstrated highly promising results. Mihata et al.^1^ first released the result of SCR using fascia lata, which showed the ASES score improved from 23.5 to 92.9 points (*p* < 0.0001). Denard et al.^2^ used SCR with dermal allograft showed that VAS pain score decreased from 5.8 ± 2.2 to 1.7 ± 2.1 (*p* < 0.001), ASES from 43.6 ± 18.6 to 77.5 ± 22.0, and SSV from, 35.0 ± 19.9 to 76.3 ± 25.2, respectively, (*p* < 0.001). In this study, the VAS pain score decreased from 8.3 ± 0.7 to 1.3 ± 0.6 (*p* < 0.001), and the AESE score improved from 37.2 ± 10.0 to 80.5 ± 5.7 (*p* < 0.001). Remarkably, our results closely resemble those of the two studies mentioned above, indicating a parallel efficacy among these approaches. Although ROM improvements in the present study were not statistically significant, the qualitative assessment revealed significant reductions in pain and enhancements in shoulder functional scales, aligning with previous studies using different SCR techniques.

### Limitation

There are limitations to this study. First, the quality of LHBT cannot be controlled preoperatively. The authors only excluded patients with the absence of LHBT before the surgery. If the LHBT is present, the authors perform the same technique regardless of LHBT size or quality because the incorporation of this LHBT tissue could be potentially valuable in improving the repair construct.^26^ Second, the non-healing of the graft to the glenoid or avulsion of the origin of the long head of LHBT is difficult to view on ultrasound images. The authors only prescribe MRI follow-ups for patients who did not progress well. Third, there was no control group.

## Conclusion

The combined SCR technique using a 2 mm dermal allograft and autologous LHBT for irreparable posterosuperior RCTs provides significant mean improvements in the patient-reported outcomes.

## Declaration of competing interest

The authors declare no conflict of interest.
